# Supplementary material for: In vivo characterization of the bacterial intramembrane-cleaving protease RseP using the heme binding tag-based assay iCliPSpy
Source: Commun Biol. 2023 Mar 18;6:287. doi: 10.1038/s42003-023-04654-z (PMC10024687; doi:10.1038/s42003-023-04654-z)
Supplement: Supplementary file 2 — Supplementary Information [file 42003_2023_4654_MOESM2_ESM.pdf]

Supplementary information

In vivo characterization of the bacterial intramembrane-cleaving protease RseP using the heme binding tag-based assay iCliPSpy

Thomas Kupke[1*], Rabea M. Götz[2], Florian M. Richter[2], Rainer Beck[1], Fabio Lolicato[1,3], Walter Nickel[1], Carsten Hopf[2], & Britta Brügger[1#]

[1]Heidelberg University Biochemistry Center (BZH), Im Neuenheimer Feld 328, 69120 Heidelberg, Germany

[2]Center for Mass Spectrometry and Optical Spectroscopy (CeMOS), Mannheim University of Applied Sciences, Paul-Wittsack-Str. 10, 68163 Mannheim, Germany

[3] Department of Physics, University of Helsinki, Helsinki, Finland

Corresponding authors

* e-mail: thomas.kupke@bzh.uni-heidelberg.de

[#] e-mail: britta.bruegger@bzh.uni-heidelberg.de

This PDF file includes:

(1) Supplementary Figures 1-7 including figure legends
(2) Supplementary Table 1: Plasmids used in this study
(3) Supplementary Table 2: Quantification of MS/MS data
(4) Supplementary Table 3: System setup for MD simulations
(5) Supplementary Note 1: Sequences of cloned DNA fragments
(6) Supplementary Note 2: Peptide analysis of MBP-TNFα fusion proteins by LC-MS/MS
(7) Supplementary Note 3: MD simulations of RseP
(8) Supplementary References

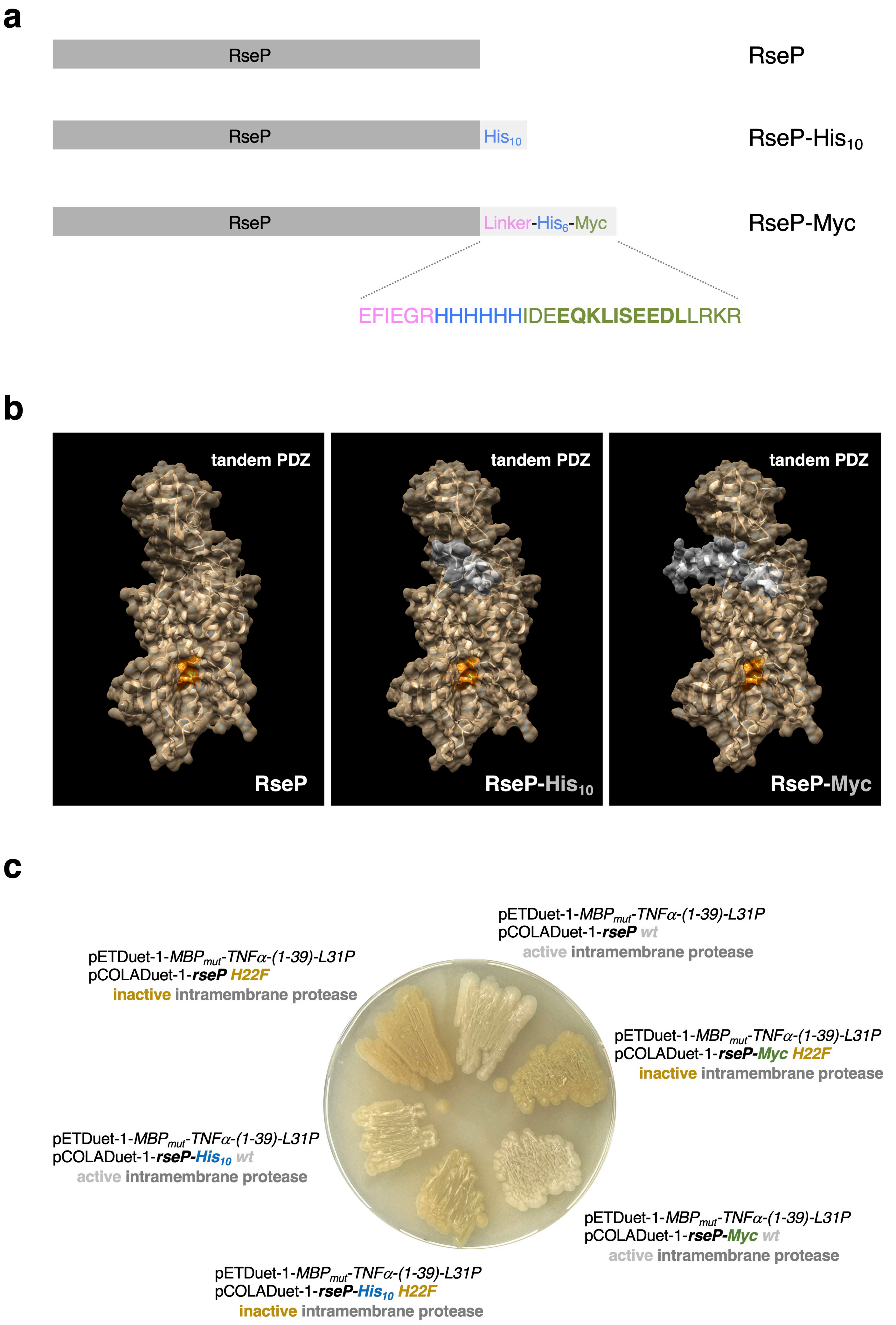

Supplementary Figure 1

Supplementary Figures 1-7 including figure legends

Supplementary Figure 1. Activity and structure of differently tagged RseP proteases. a In this study, we used untagged, C-terminally 10xHis (His_{10})-tagged and Myc-tagged RseP proteases. The Myc tag is attached to the C-terminus of RseP via a linker peptide and a 6xHis tag[1]. **b** Comparison of AlphaFold structure predictions for RseP, RseP-His_{10} and RseP-Myc. C-terminally fused peptides (shown in grey) are localized in the periplasm and can contact the PDZ tandem domains; the active site of RseP is shown in orange. **c** *E. coli* T7 Express cells harboring the two compatible plasmids pETDuet-1 and pCOLADuet-1, co-expressing genes encoding the heme binding protein MBP_{mut}-TNFα-(1-39)-L31P and active RseP wt or inactive RseP H22F (either untagged, Myc-tagged or His_{10}-tagged) intramembrane-cleaving protease, were incubated on an agar plate for five days at room temperature. Judging from the color of the cells, the His_{10} tag reduces the cleavage of MBP_{mut}-TNFα-(1-39)-L31P either by reducing the specific activity of RseP or by reducing the amount of correctly folded RseP.

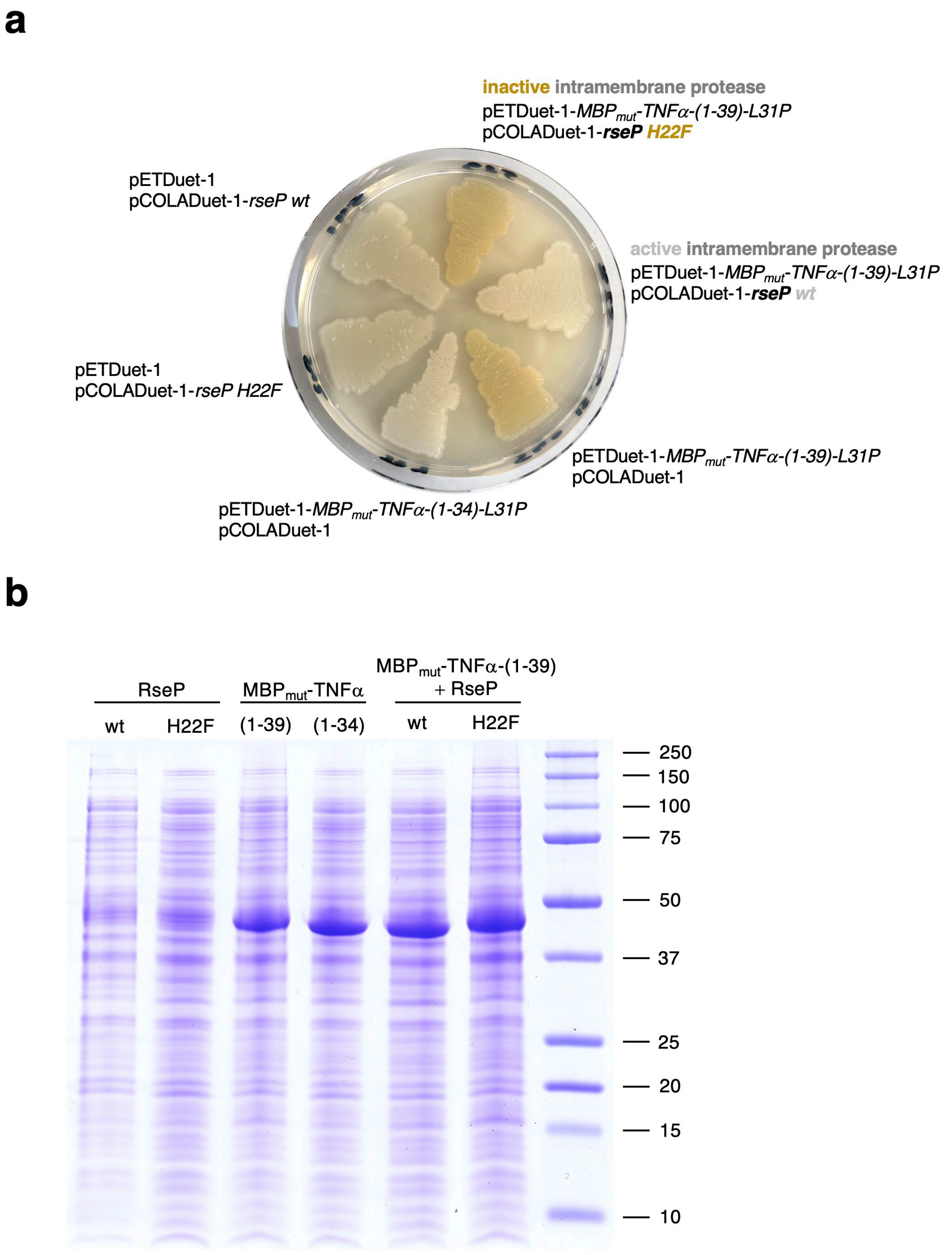

Supplementary Figure 2

Supplementary Figure 2. Visualization of *in vivo* intramembrane proteolysis catalyzed by untagged RseP. a *E. coli* T7 Express cells harboring the two compatible plasmids pETDuet-1 and pCOLADuet-1 co-expressing genes encoding the heme binding protein MBP_{mut}-TNFα-(1-39)-L31P and active RseP wt or inactive RseP H22F intramembrane-cleaving protease, respectively, were incubated on an agar plate for three days at room temperature. Control cells expressed only one gene encoding either RseP wt, RseP H22F, the substrate MBP_{mut}-TNFα-(1-39)-L31P or the putative proteolysis product MBP_{mut}-TNFα-(1-34)-L31P. **b** SDS-PAGE analysis of *E. coli* T7 Express cells expressing genes for RseP wt, RseP H22F, MBP_{mut}-TNFα-(1-39)-L31P, MBP_{mut}-TNFα-(1-34)-L31P or co-expressing genes for MBP_{mut}-TNFα-(1-39)-L31P and RseP wt or H22F, respectively, grown on a selection plate. Comparative *in vivo* analysis of RseP activities, as shown in **a,** was repeated once, and a representative result is shown. SDS-PAGE analysis of the cells was performed for only one of the two experiments. The comparative *in vivo* analysis of RseP wt and RseP H22F activities was done at least five times, and the same result was always obtained, as shown in upper right part of **a**.

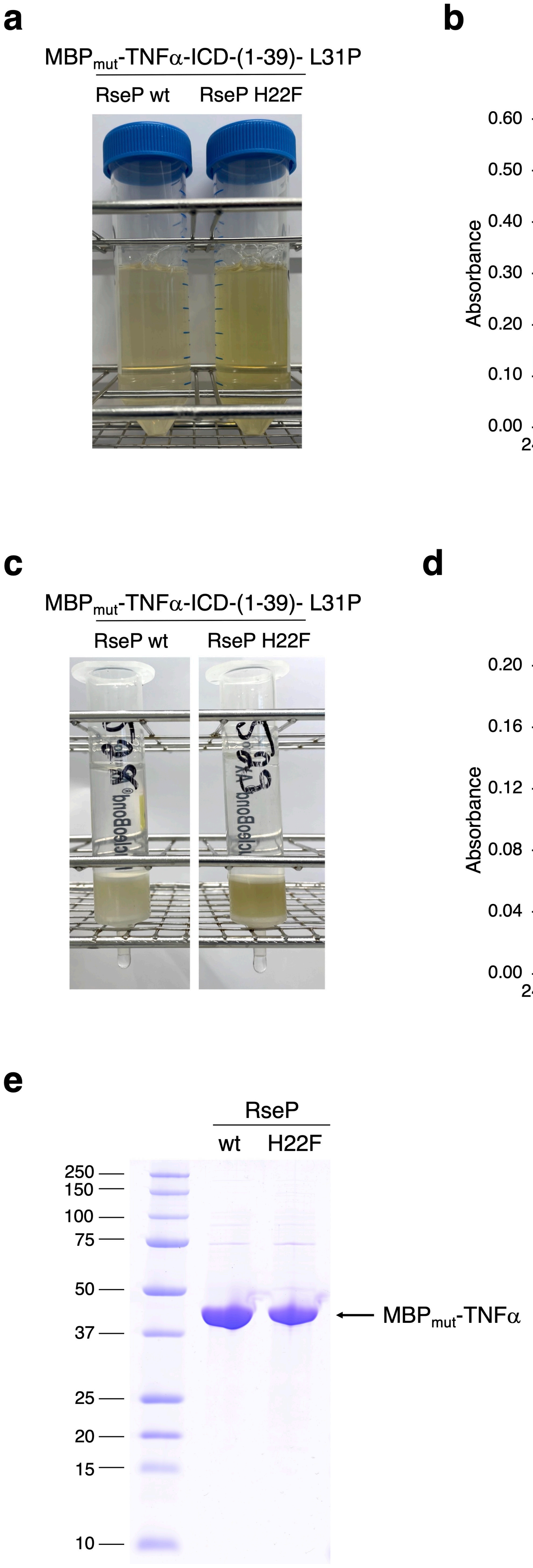

Supplementary Figure 3

Supplementary Figure 3. Analysis of cytoplasmic MBP$_{mut}$-TNFα fusion proteins processed by membrane-bound RseP. a Cytoplasmic fractions of IPTG-induced T7 cells co-expressing either genes for MBP$_{mut}$-TNFα-(1-39)-L31P and RseP wt (left tube) or genes for MBP$_{mut}$-TNFα-(1-39)-L31P and mutant RseP H22F (right tube). **b** UV/Vis spectra of cytoplasmic fractions of IPTG-induced T7 cells shown in a. Presence of unprocessed MBP$_{mut}$-TNFα-(1-39)-L31P protein (orange trace) leads to increased absorbances at 370 nm and 450 nm. **c** Purification of soluble MBP$_{mut}$-TNFα fusion proteins by amylose affinity chromatography (using TN buffer without detergent) from cytoplasmic fractions of IPTG-induced T7 cells shown in a. The purified MBP$_{mut}$-TNFα fusion proteins (which correspond to product and substrate of the RseP reaction) were then analyzed **d** by UV/Vis-spectroscopy (blue trace, + RseP wt; orange trace, + RseP H22F) and **e** by SDS-PAGE.

a

Empty vector control

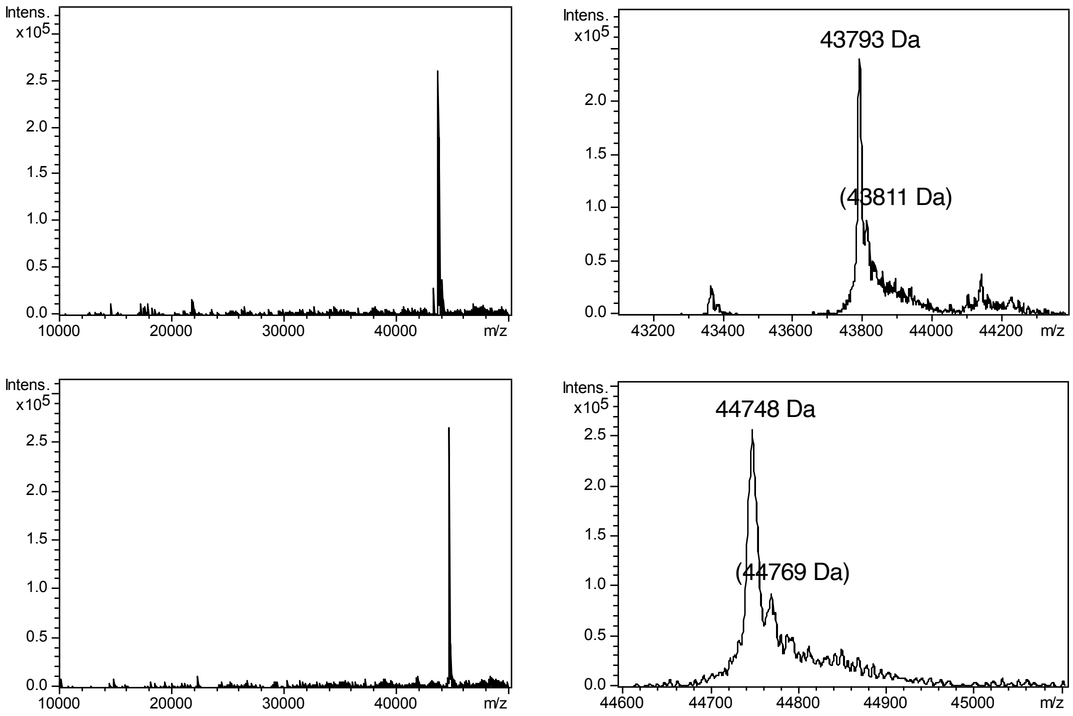

Retention time 5.4 – 5.7 min

Retention time 5.8 – 6.2 min

Supplementary Figure 4

b

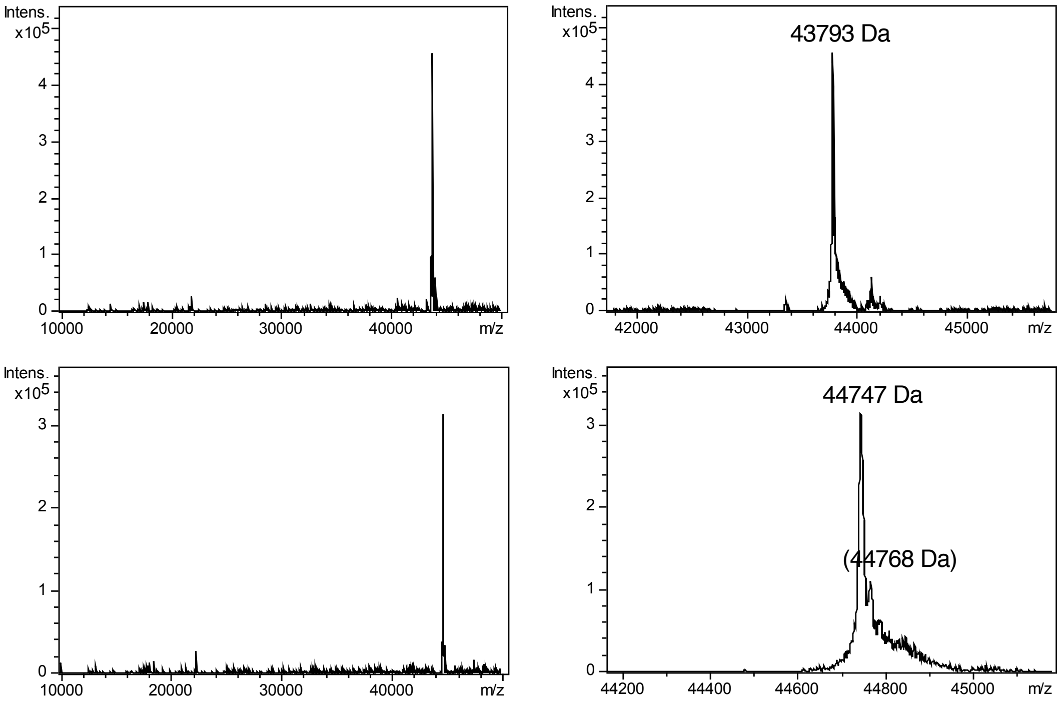

RseP-Myc H22F

Retention time 5.5 – 5.8 min

Retention time 5.9 – 6.2 min

c

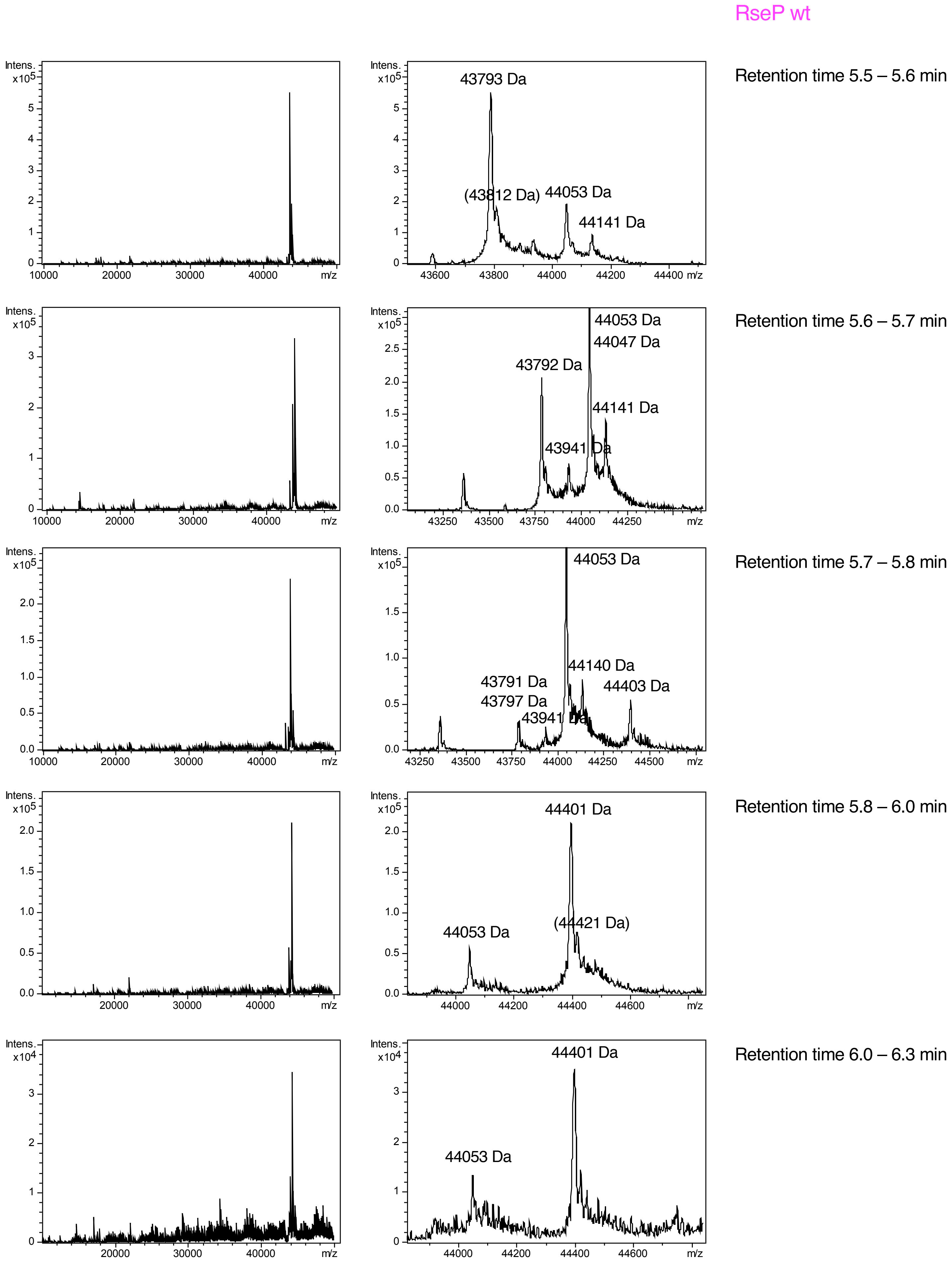
RseP wt
Retention time 5.5 – 5.6 min
43793 Da
(43812 Da)
44053 Da
44141 Da
Retention time 5.6 – 5.7 min
44053 Da
44047 Da
43792 Da
44141 Da
43941 Da
Retention time 5.7 – 5.8 min
44053 Da
44140 Da
43791 Da
43797 Da
44403 Da
43941 Da
Retention time 5.8 – 6.0 min
44401 Da
44053 Da
(44421 Da)
Retention time 6.0 – 6.3 min
44401 Da
44053 Da
Intens.
x10^5
x10^4
m/z

d

RseP-Myc wt

Retention time 5.4 – 5.7 min

Retention time 5.8 – 6.0 min

Retention time 6.0 – 6.3 min

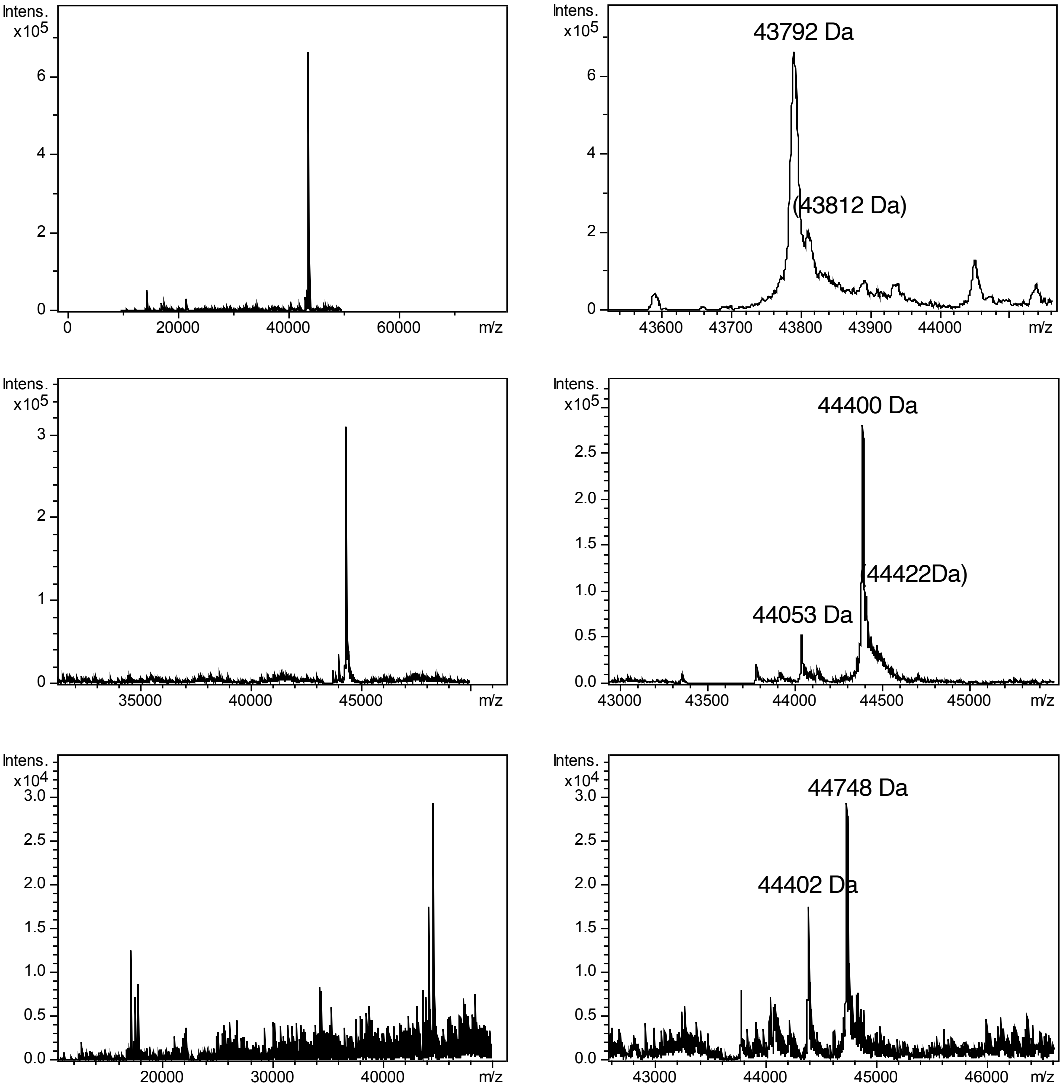

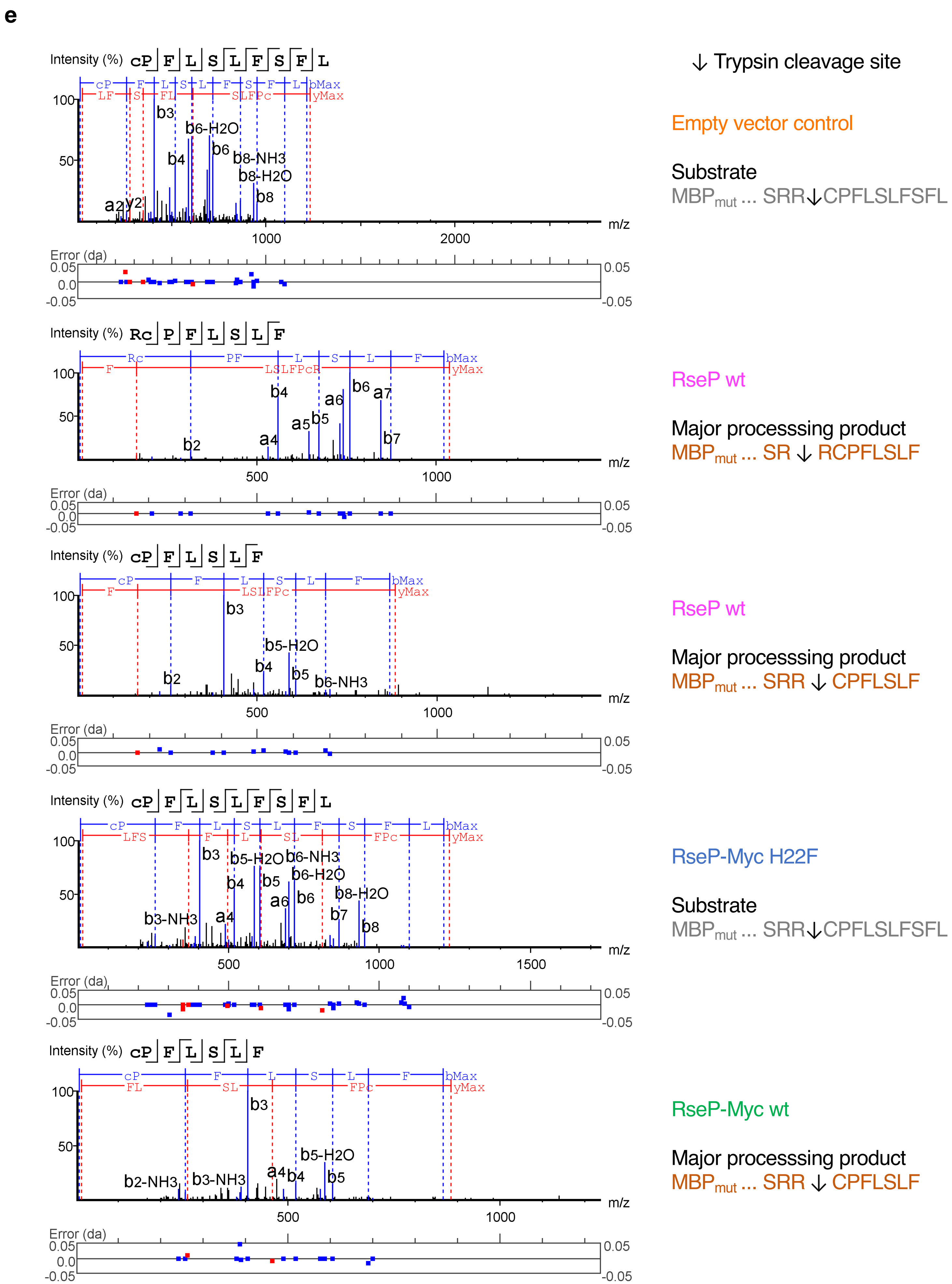

MS/MS spectra of tryptic fragments

Supplementary Figure 4. Mass spectra of substrate MBP$_{mut}$-TNFα-(1-39)-L31P and its RseP processing products. a-d Deconvoluted ESI-MS spectra (left panels, full *m/z* range; right panels, sections of full range spectra) of MBP$_{mut}$-TNFα fusion proteins purified from the membrane fraction of IPTG-induced T7 Express cells using amylose affinity chromatography and then separated by liquid chromatography as shown in Figure 5. The retention times analyzed (compare Fig. 5e) are indicated on the right and masses of putative sodium adducts are shown in brackets. *MBP$_{mut}$-TNFα-(1-39)-L31P* was co-expressed either with **a** empty vector control pCOLADuet-1, **b** pCOLADuet-1-*rseP-Myc H22F*, **c** pCOLADuet-1-*rseP wt*, or **d** with pCOLADuet-1-*rseP-Myc wt*. **e** Exemplary MS/MS spectra of C-terminal peptides of the substrate MBP$_{mut}$- … SRRCPFLSLFSFL^{39} and RseP processing product MBP$_{mut}$- … SRRCPFLSLF^{36} obtained by tryptic cleavage of the purified MBP$_{mut}$-TNFα fusion proteins are shown. Carbamidomethylated (+ 57.02 Da) cysteine residues are indicated with c (instead of C for the unmodified cysteine residue). This peptide analysis confirms the results of the intact mass analysis (a-d and Fig. 5) by sequence information.

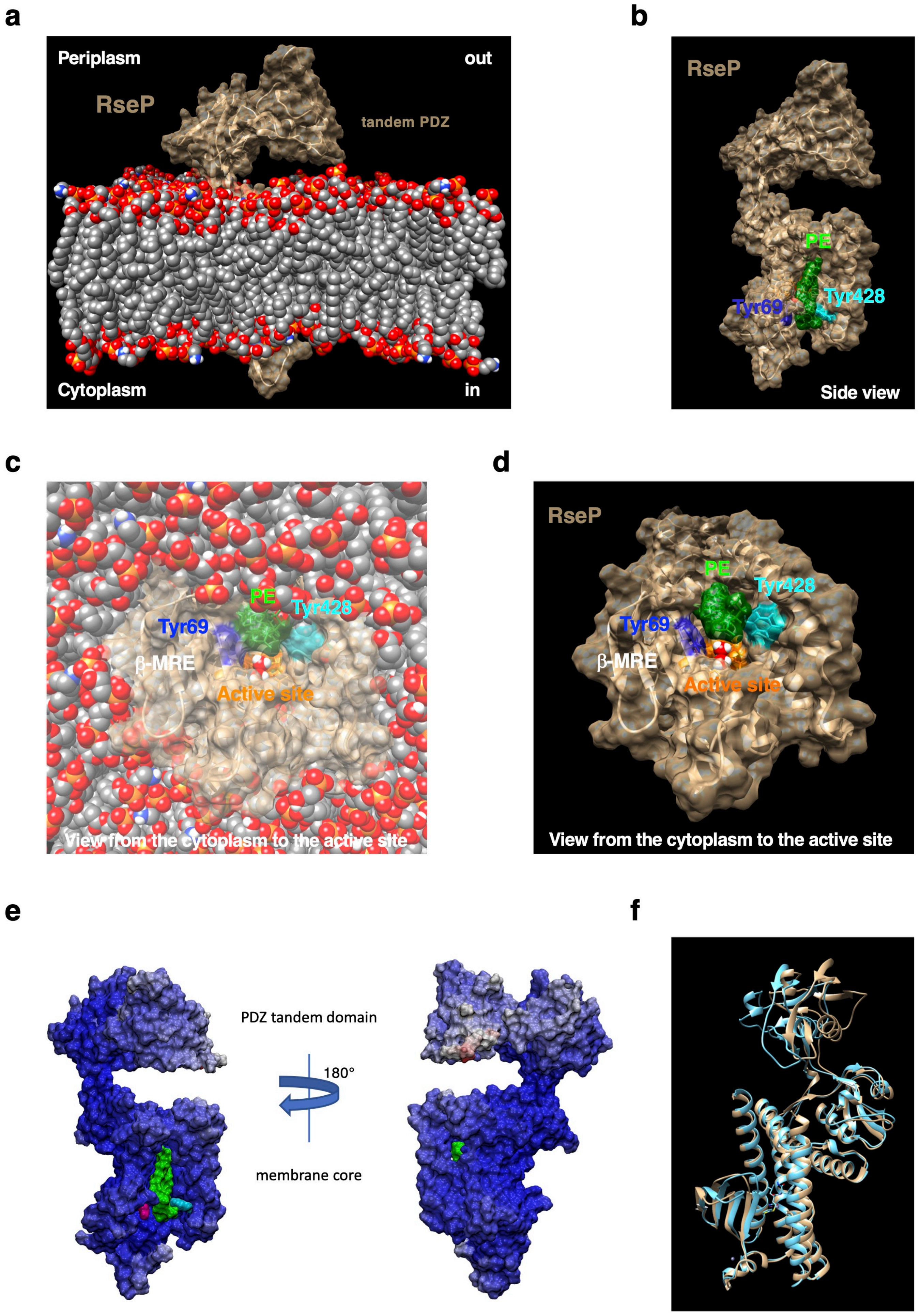

Supplementary Figure 5

Supplementary Figure 5. Molecular dynamics simulations of the AlphaFold RseP wt structure. For MD simulations of the AlphaFold RseP wt structure in a membrane environment, the Zn^{2+} ion was manually curated into the active site of RseP. The middle structures of the main cluster (see Extended methods) are visualized with Chimera. **a** Structure of full-length membrane-embedded RseP (periplasmic PDZ domain and small cytoplasmic domain are represented in the tan-colored transparent surface). The membrane molecules are shown in the sphere representation colored by element. **b** Same protein orientation as in **a** but membrane lipids are omitted in this subfigure, except for the PE lipid 1-hexadecanoyl-2-(9Z-hexadecenoyl)-*sn*-glycero-3-phosphoethanolamine (PYPE, highlighted in green), which is located next to/within the binding groove. Most of the active site (orange) is hidden by this lipid. **c, d** View from the cytoplasm to the active site [composed of His22, His26 and Asp402 complexing the Zn^{2+} ion (shown in black, but largely hidden by two water molecules) and the residues Glu23 and Asn394] shown in orange. Tyr69 at the cytosol/membrane interface is shown in dark blue, Tyr428 in cyan; β-MRE, membrane-reentrant β-loop. Tyr69 is suggested to play a role in binding the substrate peptide through hydrophobic interactions. Membrane lipids are omitted in subfigure **d,** which is also shown in Figure 6 of the main text. **e** Flexibility of the periplasmic PDZ tandem domain of RseP. The coloring of the protein structure reflects the computed root mean square fluctuation (RMSF, *i.e.* standard deviation) of atomic positions of the protein residues in the trajectory obtained after MD simulations of RseP embedded in a membrane. A BWR (blue white red) color scale was applied to illustrate regions of low fluctuations [dark blue, see the active site where the lipid PYPE (in green) is located] and higher fluctuation (light blue with a transition to white) in the more flexible tandem PDZ domain. The lipid PYPE hides most of the active site, residues Tyr69 and Tyr428 are shown in magenta and cyan, respectively. Left panel, side view as shown in b, right panel, rotated 180 degrees about Y axis. **f** Comparison of the AlphaFold predicted structure of RseP (tan; AF-P0AEH1-F1-model v1, date 2021-09-21) with the crystal structure of RseP (blue; pdb file 7W6X with bound Zn^{2+} ions in grey and bound inhibitor batimastat, released on 2022-09-07). The structure of the membrane core harbouring the active site of RseP is very similar in both

cases. However, when comparing the structures, one of two PDZ domains is rotated by a few degrees, probably due to flexibility of the PDZ tandem domain.

a

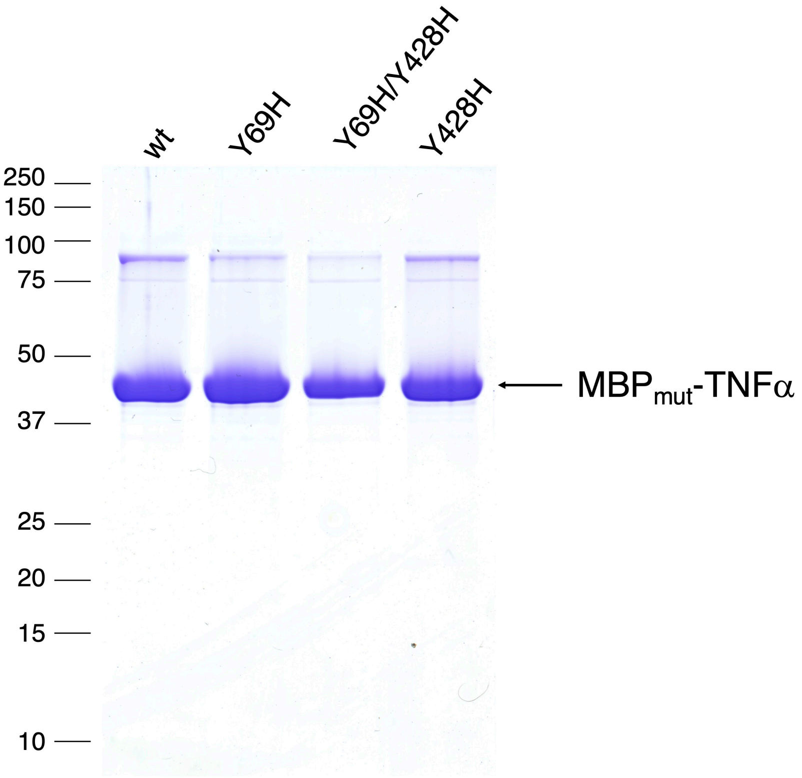
wt
Y69H
Y69H/Y428H
Y428H
250
150
100
75
50
37
25
20
15
10
MBPmut-TNFα

b

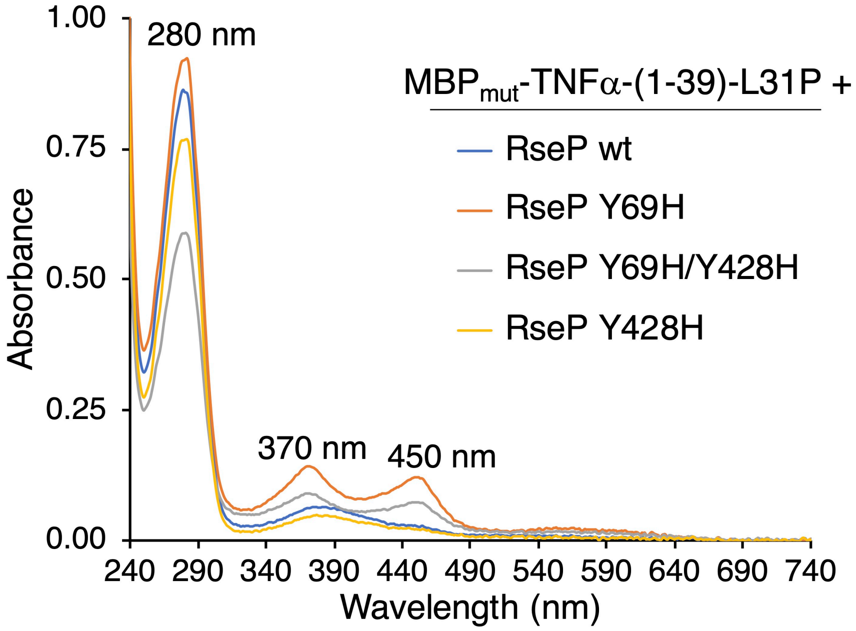
1.00
0.75
0.50
0.25
0.00
Absorbance
280 nm
370 nm
450 nm
MBPmut-TNFα-(1-39)-L31P +
RseP wt
RseP Y69H
RseP Y69H/Y428H
RseP Y428H
240 290 340 390 440 490 540 590 640 690 740
Wavelength (nm)

Supplementary Figure 6. Characterization of mutant MBP$_{mut}$-TNFα-(1-39)-L31P proteins. Purification of membrane-bound MBP$_{mut}$-TNFα fusion proteins by amylose affinity chromatography from IPTG-induced T7 cells co-expressing *MBP$_{mut}$-TNFα-(1-39)-L31P* and *rseP wt*, *rseP Y69H*, *rseP Y69H/Y428H* and *rseP Y428H*, respectively, was analyzed by **a** SDS-PAGE and **b** by UV/Vis spectroscopy (blue trace, + RseP wt; red trace, + RseP Y69H; grey trace, + RseP Y69H/Y428H; orange trace, RseP Y428H). The comparative MBP$_{mut}$-TNFα purification shown in **a** and **b** was performed once, but the comparative purification was repeated for RseP wt and RseP Y69H and in a second experiment for RseP Y69H/Y428H and RseP Y428H, respectively. **b** shows representative UV/Vis spectra.

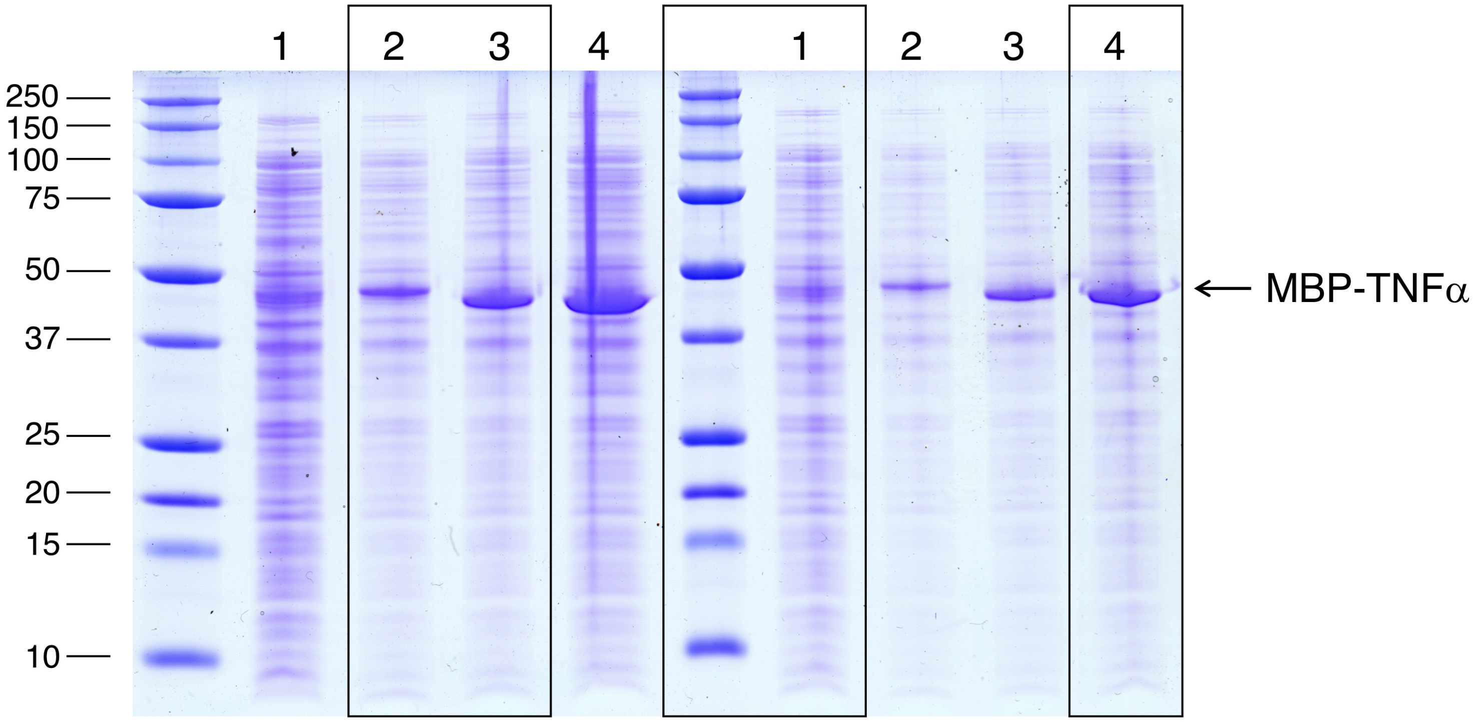

Supplementary Figure 7

Supplementary Figure 7. The framed lanes of this gel were used for Fig. 2c.

Supplementary Table 1: Plasmids used in this study

Plasmid name	Description
pTK1050	pETDuet-1-*His-MBP-TNF*α*-(1-39)-L31P*
pTK1130	pETDuet-1-*MBP*$_{mut}$*-TNF*α*-(1-39)-L31P*
pTK1150	pETDuet-1-*MBP*$_{mut}$*-TNF*α*-(1-34)-L31P*
pTK1121	pCOLADuet-1-*rseP wt*
pTK1134	pCOLADuet-1-*rseP H22F*
pTK1178	pCOLADuet-1-*rseP-His*$_{10}$ *wt*
pTK1180	pCOLADuet-1-*rseP-His*$_{10}$ *H22F*
pTK1232	pCOLADuet-1-*rseP G1*
pTK1240	pCOLADuet-1-*rseP Y69H*
pTK1248	pCOLADuet-1-*rseP Y69H/Y428H*
pTK1250	pCOLADuet-1-*rseP Y428H*
pTK1299	pCOLADuet-1-*rseP-Myc wt*
pTK1300	pCOLADuet-1-*rseP-Myc H22F*

Supplementary Table 2: Quantification of MS/MS data

Sequence	Tryptic peptides	Sum Percent of total C-term Area			
		RseP-Myc wt	RseP-Myc H22F	RseP wt	Empty vector control
... CPFLSLFSFL	RC(+57.02)PFLSLFSFL<br>RC(+58.01)PFLSLFSFL<br>C(+57.02)PFLSLFSFL<br>C(+57.02)PFLSLFSFL(+21.98)<br>LSLFSFL<br>C(+42.01)(-2.02)PFLSLFSFL	**26.07%**	**100.00%**	**5.09%**	**96.60%**
**... CPFLSLFSF**	C(+57.02)PFLSLFSF	0.00%	0.00%	0.00%	0.25%
**... CPFLSLFS**	RC(+57.02)PFLSLFS	0.00%	0.00%	1.94%	0.00%
**... CPFLSLF**	RC(+57.02)PFLSLF<br>RC(+58.01)PFLSLF<br>C(+57.02)PFLSLF	**73.93%**	**0.00%**	**92.97%**	**3.15%**

Comment: This table gives an incomplete overview of the quantification of RseP cleavage products, as small tryptic fragments (for example the peptides RcPFL and cPFL obtained from the second major cleavage product of RseP, MBP_{mut}- … CPFL[33]) were not detectable by LC-MS/MS. However, the results confirm that the reporter protein MBP_{mut}- … C[3]PFLSLFSFL[39] is mostly processed in the presence of overproduced active RseP and that MBP_{mut}- … SRRCPFLSLF[36] (but neither MBP_{mut}- … CPFLSLFSF[38] nor MBP_{mut}- … CPFLSLFS[37]) is a major processing product (as has also been shown by intact mass analysis, Fig. 5e).

Supplementary Table 3: System setup for MD simulations

simulation box dimensions	10x10x14 nm
total number of atoms:	~150000*
water molecules	~35500*
DMPE	12
PYPE	24
TYCL2	37
DPPE	12
PMCL2	97
sodium chloride concentration	100 mM

* The total number of atoms and water molecules slightly differs between each independent repetition.

Supplementary Note 1: Sequences of cloned DNA fragments

5´ and 3´ restriction sites (*Nco*I and *Xho*I sites, respectively) of the shown inserts of pETDuet-1 and pCOLADuet-1 expression vectors are underlined, start and stop codons are in bold black letters.

pTK1050 = pETDuet-1-*His-MBP-TNFα-(1-39)-L31P*

CC**ATG**GATCATCATCACCACCATCACCATCATCATCACAAAATTGAAGAAGGCAAACTGGTCATTTGGATC
AATGGTGATAAAGGCTATAATGGTCTGGCAGAAGTTGGCAAAAAATTCGAAAAAGATACCGGCATTAAAGT
GACCGTTGAACATCCGGATAAACTGGAAGAAAAATTTCCGCAGGTTGCAGCAACCGGTGATGGTCCGGATA
TTATCTTTTGGGCACATGATCGTTTTGGTGGTTATGCACAGAGCGGTCTGCTGGCAGAAATTACACCGGAT
AAAGCATTTCAGGACAAACTGTATCCGTTTACCTGGGATGCAGTTCGCTATAACGGTAAACTGATTGCATA
TCCGATTGCAGTTGAAGCACTGAGCCTGATCTATAACAAAGATCTGCTGCCGAATCCGCCTAAAACCTGGG
AAGAAATTCCGGCACTGGATAAAGAACTGAAAGCAAAAGGTAAAAGCGCACTGATGTTTAATCTGCAAGAA
CCGTATTTTACCTGGCCTCTGATTGCAGCAGATGGTGGCTATGCATTCAAATATGAAAACGGCAAATACGA
TATCAAGGATGTTGGTGTTGATAATGCGGGTGCAAAAGCCGGTCTGACCTTTCTGGTTGATCTGATCAAAA
ACAAACACATGAATGCCGATACCGATTATAGCATTGCAGAAGCAGCATTTAACAAAGGTGAAACCGCAATG
ACAATTAATGGTCCGTGGGCATGGTCAAATATTGATACCAGCAAAGTGAATTATGGTGTTACCGTTCTGCC
GACATTTAAAGGTCAGCCGAGCAAACCGTTTGTTGGTGTGCTGAGCGCAGGTATTAATGCAGCAAGCCCGA
ACAAAGAACTGGCAAAAGAATTTCTGGAAAACTATCTGCTGACCGATGAAGGTCTGGAAGCAGTGAATAAA
GATAAACCGCTGGGTGCAGTTGCACTGAAAAGCTATGAAGAAGAACTGGTTAAAGATCCGCGTATTGCAGC
CACAATGGAAAATGCACAGAAAGGCGAAATTATGCCGAATATTCCGCAGATGAGCGCATTTTGGTATGCCG
TTCGTACCGCAGTGATTAATGCCGCATCAGGTCGTCAGACCGTTGATGCAGCACTGGCAGCAGCACAGACC
AATGCAGCAGCAATGAGCACCGAAAGCATGATTCGTGATGTTGAACTGGCCGAAGAAGCACTGCCGAAAAA
AACCGGTGGTCCGCAGGGTAGCCGTCGTTGTCCGTTTCTGAGCCTGTTTAGCTTTCTG**TAATAA**CTCGAG

pTK1130 = pETDuet-1-*MBP_{mut}-TNFα-(1-39)-L31P*

CC**ATG**GTGAAAATCGAAGAAGGCAAACTGGTTATTTGGATCAATGGCGATAAAGGCTATAATGGTCTGGCA
GAAGTTGGCAAAAAATTCGAAAAAGATACCGGCATTAAAGTGACCGTTGAACATCCGGATAAACTGGAAGA
AAAATTTCCGCAGGTTGCAGCAACCGGTGATGGTCCGGATATTATCTTTTGGGCACATGATCGTTTTGGTG
GTTATGCACAGAGCGGTCTGCTGGCAGAAATTACACCGGCAGCAGCATTTCAGGACAAACTGTATCCGTTT
ACCTGGGATGCAGTTCGCTATAACGGTAAACTGATTGCATATCCGATTGCAGTTGAAGCACTGAGCCTGAT
CTATAACAAAGATCTGCTGCCGAATCCGCCTAAAACCTGGGAAGAAATTCCGGCACTGGATAAAGAACTGA
AAGCAAAAGGTAAAAGCGCACTGATGTTTAATCTGCAAGAACCGTATTTTACCTGGCCTCTGATTGCAGCA
GATGGTGGCTATGCATTCAAATATGCAGCAGGCAAATATGACATTAAAGATGTTGGTGTTGATAATGCGGG
TGCAAAAGCCGGTCTGACCTTTCTGGTTGATCTGATCAAAAACAAACACATGAATGCCGATACCGATTATA
GCATTGCAGAACATGCATTTAATCATGGCGAAACCGCAATGACAATTAATGGTCCGTGGGCATGGTCAAAT
ATTGATACCAGCGCAGTTAATTATGGTGTTACCGTTCTGCCGACATTTAAAGGTCAGCCGAGCAAACCGTT
TGTTGGTGTGCTGAGCGCAGGTATTAATGCAGCAAGCCCGAACAAAGAACTGGCAAAAGAATTTCTGGAAA
ACTATCTGCTGACCGATGAAGGTCTGGAAGCAGTGAATAAAGATAAACCGCTGGGTGCAGTTGCACTGAAA
AGCTATGAAGAAGAACTGGTTAAAGATCCGCGTGTTGCAGCCACAATGGAAAATGCACAGAAAGGTGAAAT
TATGCCGAATATTCCGCAGATGAGCGCATTTTGGTATGCCGTTCGTACCGCAGTGATTAATGCCGCATCAG
GTCGTCAGACCGTTGATGCAGCACTGGCAGCAGCCCAGACCAATGCAGCAGCAATGAGCACCGAAAGCATG
ATTCGTGATGTTGAACTGGCCGAAGAAGCACTGCCGAAAAAAACCGGTGGTCCGCAGGGTAGCCGTCGTTG
TCCGTTTCTGAGCCTGTTTAGCTTTCTG**TAATAA**CTCGAG

pTK1150 = pETDuet-1-*MBP_{mut}-TNFα-(1-34)-L31P*

CC**ATG**GTGAAAATCGAAGAAGGCAAACTGGTTATTTGGATCAATGGCGATAAAGGCTATAATGGTCTGGCA
GAAGTTGGCAAAAAATTCGAAAAAGATACCGGCATTAAAGTGACCGTTGAACATCCGGATAAACTGGAAGA
AAAATTTCCGCAGGTTGCAGCAACCGGTGATGGTCCGGATATTATCTTTTGGGCACATGATCGTTTTGGTG
GTTATGCACAGAGCGGTCTGCTGGCAGAAATTACACCGGCAGCAGCATTTCAGGACAAACTGTATCCGTTT
ACCTGGGATGCAGTTCGCTATAACGGTAAACTGATTGCATATCCGATTGCAGTTGAAGCACTGAGCCTGAT
CTATAACAAAGATCTGCTGCCGAATCCGCCTAAAACCTGGGAAGAAATTCCGGCACTGGATAAAGAACTGA
AAGCAAAAGGTAAAAGCGCACTGATGTTTAATCTGCAAGAACCGTATTTTACCTGGCCTCTGATTGCAGCA
GATGGTGGCTATGCATTCAAATATGCAGCAGGCAAATATGACATTAAAGATGTTGGTGTTGATAATGCGGG

TGCAAAAGCCGGTCTGACCTTTCTGGTTGATCTGATCAAAAACAAACACATGAATGCCGATACCGATTATA
GCATTGCAGAACATGCATTTAATCATGGCGAAACCGCAATGACAATTAATGGTCCGTGGGCATGGTCAAAT
ATTGATACCAGCGCAGTTAATTATGGTGTTACCGTTCTGCCGACATTTAAAGGTCAGCCGAGCAAACCGTT
TGTTGGTGTGCTGAGCGCAGGTATTAATGCAGCAAGCCCGAACAAAGAACTGGCAAAAGAATTTCTGGAAA
ACTATCTGCTGACCGATGAAGGTCTGGAAGCAGTGAATAAAGATAAACCGCTGGGTGCAGTTGCACTGAAA
AGCTATGAAGAAGAACTGGTTAAAGATCCGCGTGTTGCAGCCACAATGGAAAATGCACAGAAAGGTGAAAT
TATGCCGAATATTCCGCAGATGAGCGCATTTTGGTATGCCGTTCGTACCGCAGTGATTAATGCCGCATCAG
GTCGTCAGACCGTTGATGCAGCACTGGCAGCAGCCCAGACCAATGCAGCAGCAATGAGCACCGAAAGCATG
ATTCGTGATGTTGAACTGGCCGAAGAAGCACTGCCGAAAAAAACCGGTGGTCCGCAGGGTAGCCGTCGTTG
TCCGTTTCTGAGC**TAATAA**CTCGAG

pTK1121 = pCOLADuet-1-*rseP wt*
CC**ATG**GTGAGCTTTCTGTGGGATTTAGCCAGCTTTATTGTTGCACTGGGTGTTCTGATTACCGTGCATGAA
TTTGGTCATTTTTGGGTTGCACGTCGTTGTGGTGTTCGTGTTGAACGTTTTAGCATTGGTTTTGGTAAAGC
ACTGTGGCGTCGTACCGATAAACTGGGCACCGAATATGTTATTGCACTGATTCCGTTAGGTGGCTATGTTA
AAATGCTGGATGAACGTGCAGAACCGGTTGTGCCGGAACTGCGTCATCATGCATTTAACAATAAAAGCGTT
GGTCAGCGTGCAGCAATTATTGCAGCAGGTCCGGTTGCCAATTTTATCTTTGCAATTTTTGCCTACTGGCT
GGTGTTTATTATCGGTGTTCCGGGTGTTCGTCCGGTTGTTGGTGAAATTGCAGCAAATAGCATTGCAGCCG
AAGCACAGATTGCACCGGGTACAGAACTGAAAGCAGTTGATGGTATTGAAACACCGGATTGGGATGCAGTT
CGTCTGCAGCTGGTTGATAAAATCGGTGATGAAAGCACCACCATTACCGTTGCACCGTTTGGTAGCGATCA
GCGTCGTGATGTTAAACTGGATCTGCGTCATTGGGCATTTGAACCGGATAAAGAAGATCCGGTTAGCAGCC
TGGGTATTCGTCCGCGTGGTCCGCAGATTGAACCGGTGCTGGAAAATGTTCAGCCGAATAGCGCAGCAAGC
AAAGCAGGTCTGCAGGCAGGCGATCGTATTGTGAAAGTGGATGGTCAGCCGCTGACACAGTGGGTTACCTT
TGTTATGCTGGTTCGTGATAATCCGGGTAAAAGCCTGGCACTGGAAATTGAACGTCAGGGTAGTCCGCTGA
GTCTGACCCTGATTCCGGAAAGCAAACCTGGTAATGGTAAAGCGATTGGCTTTGTGGGTATTGAACCGAAA
GTTATTCCGCTGCCGGATGAATATAAAGTTGTTCGTCAGTATGGTCCGTTTAACGCAATTGTTGAAGCAAC
CGATAAAACCTGGCAGCTGATGAAACTGACCGTTAGCATGCTGGGTAAACTGATTACAGGTGACGTGAAAC
TGAATAATCTGAGCGGTCCGATTAGCATTGCCAAAGGTGCAGGTATGACCGCAGAACTGGGCGTTGTTTAT
TACCTGCCGTTTCTGGCACTGATTAGCGTTAATCTGGGCATTATTAACCTGTTTCCACTGCCGGTTCTGGA
TGGTGGTCATCTGCTGTTTTTAGCCATCGAAAAAATCAAAGGTGGTCCGGTGAGCGAACGTGTTCAGGATT
TTTGTTATCGTATTGGTAGCATTCTGCTGGTTCTGCTGATGGGTTTAGCACTGTTTAATGATTTTAGCCGT
CTG**TAATAA**CTCGAG

pTK1134 = pCOLADuet-1-*rseP H22F*
CC**ATG**GTGAGCTTTCTGTGGGATTTAGCCAGCTTTATTGTTGCACTGGGTGTTCTGATTACCGTGTTTGAA
TTTGGTCATTTTTGGGTTGCACGTCGTTGTGGTGTTCGTGTTGAACGTTTTAGCATTGGTTTTGGTAAAGC
ACTGTGGCGTCGTACCGATAAACTGGGCACCGAATATGTTATTGCACTGATTCCGTTAGGTGGCTATGTTA
AAATGCTGGATGAACGTGCAGAACCGGTTGTGCCGGAACTGCGTCATCATGCATTTAACAATAAAAGCGTT
GGTCAGCGTGCAGCAATTATTGCAGCAGGTCCGGTTGCCAATTTTATCTTTGCAATTTTTGCCTACTGGCT
GGTGTTTATTATCGGTGTTCCGGGTGTTCGTCCGGTTGTTGGTGAAATTGCAGCAAATAGCATTGCAGCCG
AAGCACAGATTGCACCGGGTACAGAACTGAAAGCAGTTGATGGTATTGAAACACCGGATTGGGATGCAGTT
CGTCTGCAGCTGGTTGATAAAATCGGTGATGAAAGCACCACCATTACCGTTGCACCGTTTGGTAGCGATCA
GCGTCGTGATGTTAAACTGGATCTGCGTCATTGGGCATTTGAACCGGATAAAGAAGATCCGGTTAGCAGCC
TGGGTATTCGTCCGCGTGGTCCGCAGATTGAACCGGTGCTGGAAAATGTTCAGCCGAATAGCGCAGCAAGC
AAAGCAGGTCTGCAGGCAGGCGATCGTATTGTGAAAGTGGATGGTCAGCCGCTGACACAGTGGGTTACCTT
TGTTATGCTGGTTCGTGATAATCCGGGTAAAAGCCTGGCACTGGAAATTGAACGTCAGGGTAGTCCGCTGA
GTCTGACCCTGATTCCGGAAAGCAAACCTGGTAATGGTAAAGCGATTGGCTTTGTGGGTATTGAACCGAAA
GTTATTCCGCTGCCGGATGAATATAAAGTTGTTCGTCAGTATGGTCCGTTTAACGCAATTGTTGAAGCAAC
CGATAAAACCTGGCAGCTGATGAAACTGACCGTTAGCATGCTGGGTAAACTGATTACAGGTGACGTGAAAC
TGAATAATCTGAGCGGTCCGATTAGCATTGCCAAAGGTGCAGGTATGACCGCAGAACTGGGCGTTGTTTAT
TACCTGCCGTTTCTGGCACTGATTAGCGTTAATCTGGGCATTATTAACCTGTTTCCACTGCCGGTTCTGGA
TGGTGGTCATCTGCTGTTTTTAGCCATCGAAAAAATCAAAGGTGGTCCGGTGAGCGAACGTGTTCAGGATT
TTTGTTATCGTATTGGTAGCATTCTGCTGGTTCTGCTGATGGGTTTAGCACTGTTTAATGATTTTAGCCGT
CTG**TAATAA**CTCGAG

pTK1178 = pCOLADuet-1-*rseP-His$_{10}$ wt*
CC**ATG**GTGAGCTTTCTGTGGGATTTAGCCAGCTTTATTGTTGCACTGGGTGTTCTGATTACCGTGCATGAA
TTTGGTCATTTTTGGGTTGCACGTCGTTGTGGTGTTCGTGTTGAACGTTTTAGCATTGGTTTTGGTAAAGC

ACTGTGGCGTCGTACCGATAAACTGGGCACCGAATATGTTATTGCACTGATTCCGTTAGGTGGCTATGTTA
AAATGCTGGATGAACGTGCAGAACCGGTTGTGCCGGAACTGCGTCATCATGCATTTAACAATAAAAGCGTT
GGTCAGCGTGCAGCAATTATTGCAGCAGGTCCGGTTGCCAATTTTATCTTTGCAATTTTTGCCTACTGGCT
GGTGTTTATTATCGGTGTTCCGGGTGTTCGTCCGGTTGTTGGTGAAATTGCAGCAAATAGCATTGCAGCCG
AAGCACAGATTGCACCGGGTACAGAACTGAAAGCAGTTGATGGTATTGAAACACCGGATTGGGATGCAGTT
CGTCTGCAGCTGGTTGATAAAATCGGTGATGAAAGCACCACCATTACCGTTGCACCGTTTGGTAGCGATCA
GCGTCGTGATGTTAAACTGGATCTGCGTCATTGGGCATTTGAACCGGATAAAGAAGATCCGGTTAGCAGCC
TGGGTATTCGTCCGCGTGGTCCGCAGATTGAACCGGTGCTGGAAAATGTTCAGCCGAATAGCGCAGCAAGC
AAAGCAGGTCTGCAGGCAGGCGATCGTATTGTGAAAGTGGATGGTCAGCCGCTGACACAGTGGGTTACCTT
TGTTATGCTGGTTCGTGATAATCCGGGTAAAAGCCTGGCACTGGAAATTGAACGTCAGGGTAGTCCGCTGA
GTCTGACCCTGATTCCGGAAAGCAAACCTGGTAATGGTAAAGCGATTGGCTTTGTGGGTATTGAACCGAAA
GTTATTCCGCTGCCGGATGAATATAAAGTTGTTCGTCAGTATGGTCCGTTTAACGCAATTGTTGAAGCAAC
CGATAAAACCTGGCAGCTGATGAAACTGACCGTTAGCATGCTGGGTAAACTGATTACAGGTGACGTGAAAC
TGAATAATCTGAGCGGTCCGATTAGCATTGCCAAAGGTGCAGGTATGACCGCAGAACTGGGCGTTGTTTAT
TACCTGCCGTTTCTGGCACTGATTAGCGTTAATCTGGGCATTATTAACCTGTTTCCACTGCCGGTTCTGGA
TGGTGGTCATCTGCTGTTTTTAGCCATCGAAAAAATCAAAGGTGGTCCGGTGAGCGAACGTGTTCAGGATT
TTTGTTATCGTATTGGTAGCATTCTGCTGGTTCTGCTGATGGGTTTAGCACTGTTTAATGATTTTAGCCGT
CTGCATCATCACCACCATCACCATCATCATCAC**TAATAA**CTCGAG

pTK1180 = pCOLADuet-1-*rseP-His$_{10}$ H22F*
CC**ATG**GTGAGCTTTCTGTGGGATTTAGCCAGCTTTATTGTTGCACTGGGTGTTCTGATTACCGTGTTTGAA
TTTGGTCATTTTTGGGTTGCACGTCGTTGTGGTGTTCGTGTTGAACGTTTTAGCATTGGTTTTGGTAAAGC
ACTGTGGCGTCGTACCGATAAACTGGGCACCGAATATGTTATTGCACTGATTCCGTTAGGTGGCTATGTTA
AAATGCTGGATGAACGTGCAGAACCGGTTGTGCCGGAACTGCGTCATCATGCATTTAACAATAAAAGCGTT
GGTCAGCGTGCAGCAATTATTGCAGCAGGTCCGGTTGCCAATTTTATCTTTGCAATTTTTGCCTACTGGCT
GGTGTTTATTATCGGTGTTCCGGGTGTTCGTCCGGTTGTTGGTGAAATTGCAGCAAATAGCATTGCAGCCG
AAGCACAGATTGCACCGGGTACAGAACTGAAAGCAGTTGATGGTATTGAAACACCGGATTGGGATGCAGTT
CGTCTGCAGCTGGTTGATAAAATCGGTGATGAAAGCACCACCATTACCGTTGCACCGTTTGGTAGCGATCA
GCGTCGTGATGTTAAACTGGATCTGCGTCATTGGGCATTTGAACCGGATAAAGAAGATCCGGTTAGCAGCC
TGGGTATTCGTCCGCGTGGTCCGCAGATTGAACCGGTGCTGGAAAATGTTCAGCCGAATAGCGCAGCAAGC
AAAGCAGGTCTGCAGGCAGGCGATCGTATTGTGAAAGTGGATGGTCAGCCGCTGACACAGTGGGTTACCTT
TGTTATGCTGGTTCGTGATAATCCGGGTAAAAGCCTGGCACTGGAAATTGAACGTCAGGGTAGTCCGCTGA
GTCTGACCCTGATTCCGGAAAGCAAACCTGGTAATGGTAAAGCGATTGGCTTTGTGGGTATTGAACCGAAA
GTTATTCCGCTGCCGGATGAATATAAAGTTGTTCGTCAGTATGGTCCGTTTAACGCAATTGTTGAAGCAAC
CGATAAAACCTGGCAGCTGATGAAACTGACCGTTAGCATGCTGGGTAAACTGATTACAGGTGACGTGAAAC
TGAATAATCTGAGCGGTCCGATTAGCATTGCCAAAGGTGCAGGTATGACCGCAGAACTGGGCGTTGTTTAT
TACCTGCCGTTTCTGGCACTGATTAGCGTTAATCTGGGCATTATTAACCTGTTTCCACTGCCGGTTCTGGA
TGGTGGTCATCTGCTGTTTTTAGCCATCGAAAAAATCAAAGGTGGTCCGGTGAGCGAACGTGTTCAGGATT
TTTGTTATCGTATTGGTAGCATTCTGCTGGTTCTGCTGATGGGTTTAGCACTGTTTAATGATTTTAGCCGT
CTGCATCATCACCACCATCACCATCATCATCAC**TAATAA**CTCGAG

pTK1232 = pCOLADuet-1-*rseP G1*
CC**ATG**GTGAGCTTTCTGTGGGATTTAGCCAGCTTTATTGTTGCACTGGGTGTTCTGATTACCGTGCATGAA
TTTGGTCATTTTTGGGTTGCTCGTCGTTGTGGTGTTCGTGTTGAACGTTTTAGCATTGGTTTTGGTAAAGC
ACTGTGGCGTCGTACCGATAAACTGGGCACCGAATATGTTATTGCACTGATTCCGTTAGGTGGCCATGTTA
AAATGCTGGATGAACGTGCAGAACCGGTTGTGCCGGAACTGCGTCATCATGCATTTAACAATAAAAGCGTT
GGTCAGCGTGCAGCAATTATTGCAGCAGGTCCGGTTGCCAATTTTATCTTTGCAATTTTTGCCTACTGGCT
GGTGTTTATTATCGGTGTTCCGGGTGTTCGTCCGGTTGTTGGTGAAATTGCAGCAAATAGCATTGCAGCCG
AAGCACAGATTGCACCGGGTACAGAACTGAAAGCAGTTGATGGTATTGAAACACCGGATAGGGATGCAGTT
CGTCTGCAGCTGGTTGATAAAATCGGTGATGAAAGAGCCACCATTACCGTTGCACCGTTTGGTAGCGATCA
GCGTCGTGATGTTAAACTGGATCTGCGTCATTGGGCATTCGAACCGGATAAAGAAGATCCGGTTAGCAGCC
AGGGTATTCGTCCGCGTGGTCCGCAGATTGAACCGGTGCTGGAAAATGTTCAGCCGAATAGCGCAGCAAGC
AAAGTAGGTCTGCAGGCAGGCGATCGTATTGTGAAAGTGGATGGTCAGCCGCTGACACAGTGGGTTACCTT
TGTTATGCAGGTTCGTGATAATCCGGGTAAAAGCCTGGCACAGGAAATTGAACGTCAGGGTAGTCCGCTGA
GTCTGACCCTGGTTCCGGAAAGCAAACCTGGTAATGGTAAAGCGATTGGCTTTGAGGGTATTGAACCGAAA
GTTATTCCGCTGCCGGATGAATATAAAGTTGTTCGTCAGTATGGTCCGTTTAACGCAATTGTTGAAGCAAC
CGATAAAACCTGGCAGCTGATGAAACTGACCGTTAGCATGCTAGGTAAACTGATTACAGGTGACGTGAAAC
TGAATAATCTGAGCGGTCCGATTAGCATTGCCAAAGGTGCAGGTATGACCGCAGAACTGGGCGTTGTTTAT

TACCTGCCGTTTCTGGCACTGATTAGCGTTAATCTGGGCATTATTAACCTGTTTCCACTGCCGGTTCTGGA
TGGTGGTCATCTGCTGTTTTTAGCCATCGAAAAAATCAGAGGTGGTCCGGTGAGCGAACGTGTTCAGGATT
TTTGTTATCGTATTGGTAGCATTCTGCTGGTTCTGCTGATGGGTTTGGCACTGTTTAATGATTTTAGCCGT
CTG**TAATAA**CTCGAG

pTK1240 = pCOLADuet-1-*rseP Y69H*
CC**ATG**GTGAGCTTTCTGTGGGATTTAGCCAGCTTTATTGTTGCACTGGGTGTTCTGATTACCGTGCATGAA
TTTGGTCATTTTTGGGTTGCACGTCGTTGTGGTGTTCGTGTTGAACGTTTTAGCATTGGTTTTGGTAAAGC
ACTGTGGCGTCGTACCGATAAACTGGGCACCGAATATGTTATTGCACTGATTCCGTTAGGTGGCCATGTTA
AAATGCTGGATGAACGTGCAGAACCGGTTGTGCCGGAACTGCGTCATCATGCATTTAACAATAAAAGCGTT
GGTCAGCGTGCAGCAATTATTGCAGCAGGTCCGGTTGCCAATTTTATCTTTGCAATTTTTGCCTACTGGCT
GGTGTTTATTATCGGTGTTCCGGGTGTTCGTCCGGTTGTTGGTGAAATTGCAGCAAATAGCATTGCAGCCG
AAGCACAGATTGCACCGGGTACAGAACTGAAAGCAGTTGATGGTATTGAAACACCGGATTGGGATGCAGTT
CGTCTGCAGCTGGTTGATAAAATCGGTGATGAAAGCACCACCATTACCGTTGCACCGTTTGGTAGCGATCA
GCGTCGTGATGTTAAACTGGATCTGCGTCATTGGGCATTTGAACCGGATAAAGAAGATCCGGTTAGCAGCC
TGGGTATTCGTCCGCGTGGTCCGCAGATTGAACCGGTGCTGGAAAATGTTCAGCCGAATAGCGCAGCAAGC
AAAGCAGGTCTGCAGGCAGGCGATCGTATTGTGAAAGTGGATGGTCAGCCGCTGACACAGTGGGTTACCTT
TGTTATGCTGGTTCGTGATAATCCGGGTAAAAGCCTGGCACTGGAAATTGAACGTCAGGGTAGTCCGCTGA
GTCTGACCCTGATTCCGGAAAGCAAACCTGGTAATGGTAAAGCGATTGGCTTTGTGGGTATTGAACCGAAA
GTTATTCCGCTGCCGGATGAATATAAAGTTGTTCGTCAGTATGGTCCGTTTAACGCAATTGTTGAAGCAAC
CGATAAAACCTGGCAGCTGATGAAACTGACCGTTAGCATGCTGGGTAAACTGATTACAGGTGACGTGAAAC
TGAATAATCTGAGCGGTCCGATTAGCATTGCCAAAGGTGCAGGTATGACCGCAGAACTGGGCGTTGTTTAT
TACCTGCCGTTTCTGGCACTGATTAGCGTTAATCTGGGCATTATTAACCTGTTTCCACTGCCGGTTCTGGA
TGGTGGTCATCTGCTGTTTTTAGCCATCGAAAAAATCAAAGGTGGTCCGGTGAGCGAACGTGTTCAGGATT
TTTGTTATCGTATTGGTAGCATTCTGCTGGTTCTGCTGATGGGTTTAGCACTGTTTAATGATTTTAGCCGT
CTG**TAATAA**CTCGAG

pTK1248 = pCOLADuet-1-*rseP Y69H/Y428H*
CC**ATG**GTGAGCTTTCTGTGGGATTTAGCCAGCTTTATTGTTGCACTGGGTGTTCTGATTACCGTGCATGAA
TTTGGTCATTTTTGGGTTGCACGTCGTTGTGGTGTTCGTGTTGAACGTTTTAGCATTGGTTTTGGTAAAGC
ACTGTGGCGTCGTACCGATAAACTGGGCACCGAATATGTTATTGCACTGATTCCGTTAGGTGGCCATGTTA
AAATGCTGGATGAACGTGCAGAACCGGTTGTGCCGGAACTGCGTCATCATGCATTTAACAATAAAAGCGTT
GGTCAGCGTGCAGCAATTATTGCAGCAGGTCCGGTTGCCAATTTTATCTTTGCAATTTTTGCCTACTGGCT
GGTGTTTATTATCGGTGTTCCGGGTGTTCGTCCGGTTGTTGGTGAAATTGCAGCAAATAGCATTGCAGCCG
AAGCACAGATTGCACCGGGTACAGAACTGAAAGCAGTTGATGGTATTGAAACACCGGATTGGGATGCAGTT
CGTCTGCAGCTGGTTGATAAAATCGGTGATGAAAGCACCACCATTACCGTTGCACCGTTTGGTAGCGATCA
GCGTCGTGATGTTAAACTGGATCTGCGTCATTGGGCATTTGAACCGGATAAAGAAGATCCGGTTAGCAGCC
TGGGTATTCGTCCGCGTGGTCCGCAGATTGAACCGGTGCTGGAAAATGTTCAGCCGAATAGCGCAGCAAGC
AAAGCAGGTCTGCAGGCAGGCGATCGTATTGTGAAAGTGGATGGTCAGCCGCTGACACAGTGGGTTACCTT
TGTTATGCTGGTTCGTGATAATCCGGGTAAAAGCCTGGCACTGGAAATTGAACGTCAGGGTAGTCCGCTGA
GTCTGACCCTGATTCCGGAAAGCAAACCTGGTAATGGTAAAGCGATTGGCTTTGTGGGTATTGAACCGAAA
GTTATTCCGCTGCCGGATGAATATAAAGTTGTTCGTCAGTATGGTCCGTTTAACGCAATTGTTGAAGCAAC
CGATAAAACCTGGCAGCTGATGAAACTGACCGTTAGCATGCTGGGTAAACTGATTACAGGTGACGTGAAAC
TGAATAATCTGAGCGGTCCGATTAGCATTGCCAAAGGTGCAGGTATGACCGCAGAACTGGGCGTTGTTTAT
TACCTGCCGTTTCTGGCACTGATTAGCGTTAATCTGGGCATTATTAACCTGTTTCCACTGCCGGTTCTGGA
TGGTGGTCATCTGCTGTTTTTAGCCATCGAAAAAATCAAAGGTGGTCCGGTGAGCGAACGTGTTCAGGATT
TTTGTCATCGTATTGGTAGCATTCTGCTGGTTCTGCTGATGGGTTTAGCACTGTTTAATGATTTTAGCCGT
CTG**TAATAA**CTCGAG

pTK1250 = pCOLADuet-1-*rseP Y428H*
CC**ATG**GTGAGCTTTCTGTGGGATTTAGCCAGCTTTATTGTTGCACTGGGTGTTCTGATTACCGTGCATGAA
TTTGGTCATTTTTGGGTTGCACGTCGTTGTGGTGTTCGTGTTGAACGTTTTAGCATTGGTTTTGGTAAAGC
ACTGTGGCGTCGTACCGATAAACTGGGCACCGAATATGTTATTGCACTGATTCCGTTAGGTGGCTATGTTA
AAATGCTGGATGAACGTGCAGAACCGGTTGTGCCGGAACTGCGTCATCATGCATTTAACAATAAAAGCGTT
GGTCAGCGTGCAGCAATTATTGCAGCAGGTCCGGTTGCCAATTTTATCTTTGCAATTTTTGCCTACTGGCT
GGTGTTTATTATCGGTGTTCCGGGTGTTCGTCCGGTTGTTGGTGAAATTGCAGCAAATAGCATTGCAGCCG
AAGCACAGATTGCACCGGGTACAGAACTGAAAGCAGTTGATGGTATTGAAACACCGGATTGGGATGCAGTT
CGTCTGCAGCTGGTTGATAAAATCGGTGATGAAAGCACCACCATTACCGTTGCACCGTTTGGTAGCGATCA

GCGTCGTGATGTTAAACTGGATCTGCGTCATTGGGCATTTGAACCGGATAAAGAAGATCCGGTTAGCAGCC
TGGGTATTCGTCCGCGTGGTCCGCAGATTGAACCGGTGCTGGAAAATGTTCAGCCGAATAGCGCAGCAAGC
AAAGCAGGTCTGCAGGCAGGCGATCGTATTGTGAAAGTGGATGGTCAGCCGCTGACACAGTGGGTTACCTT
TGTTATGCTGGTTCGTGATAATCCGGGTAAAAGCCTGGCACTGGAAATTGAACGTCAGGGTAGTCCGCTGA
GTCTGACCCTGATTCCGGAAAGCAAACCTGGTAATGGTAAAGCGATTGGCTTTGTGGGTATTGAACCGAAA
GTTATTCCGCTGCCGGATGAATATAAAGTTGTTCGTCAGTATGGTCCGTTTAACGCAATTGTTGAAGCAAC
CGATAAAACCTGGCAGCTGATGAAACTGACCGTTAGCATGCTGGGTAAACTGATTACAGGTGACGTGAAAC
TGAATAATCTGAGCGGTCCGATTAGCATTGCCAAAGGTGCAGGTATGACCGCAGAACTGGGCGTTGTTTAT
TACCTGCCGTTTCTGGCACTGATTAGCGTTAATCTGGGCATTATTAACCTGTTTCCACTGCCGGTTCTGGA
TGGTGGTCATCTGCTGTTTTTAGCCATCGAAAAAATCAAAGGTGGTCCGGTGAGCGAACGTGTTCAGGATT
TTTGTCATCGTATTGGTAGCATTCTGCTGGTTCTGCTGATGGGTTTAGCACTGTTTAATGATTTTAGCCGT
CTG**TAATAA**CTCGAG

pTK1299 = pCOLADuet-1-*rseP-Myc wt*
CC**ATG**GTGAGCTTTCTGTGGGATTTAGCCAGCTTTATTGTTGCACTGGGTGTTCTGATTACCGTGCATGAA
TTTGGTCATTTTTGGGTTGCACGTCGTTGTGGTGTTCGTGTTGAACGTTTTAGCATTGGTTTTGGTAAAGC
ACTGTGGCGTCGTACCGATAAACTGGGCACCGAATATGTTATTGCACTGATTCCGTTAGGTGGCTATGTTA
AAATGCTGGATGAACGTGCAGAACCGGTTGTGCCGGAACTGCGTCATCATGCATTTAACAATAAAAGCGTT
GGTCAGCGTGCAGCAATTATTGCAGCAGGTCCGGTTGCCAATTTTATCTTTGCAATTTTTGCCTACTGGCT
GGTGTTTATTATCGGTGTTCCGGGTGTTCGTCCGGTTGTTGGTGAAATTGCAGCAAATAGCATTGCAGCCG
AAGCACAGATTGCACCGGGTACAGAACTGAAAGCAGTTGATGGTATTGAAACACCGGATTGGGATGCAGTT
CGTCTGCAGCTGGTTGATAAAATCGGTGATGAAAGCACCACCATTACCGTTGCACCGTTTGGTAGCGATCA
GCGTCGTGATGTTAAACTGGATCTGCGTCATTGGGCATTTGAACCGGATAAAGAAGATCCGGTTAGCAGCC
TGGGTATTCGTCCGCGTGGTCCGCAGATTGAACCGGTGCTGGAAAATGTTCAGCCGAATAGCGCAGCAAGC
AAAGCAGGTCTGCAGGCAGGCGATCGTATTGTGAAAGTGGATGGTCAGCCGCTGACACAGTGGGTTACCTT
TGTTATGCTGGTTCGTGATAATCCGGGTAAAAGCCTGGCACTGGAAATTGAACGTCAGGGTAGTCCGCTGA
GTCTGACCCTGATTCCGGAAAGCAAACCTGGTAATGGTAAAGCGATTGGCTTTGTGGGTATTGAACCGAAA
GTTATTCCGCTGCCGGATGAATATAAAGTTGTTCGTCAGTATGGTCCGTTTAACGCAATTGTTGAAGCAAC
CGATAAAACCTGGCAGCTGATGAAACTGACCGTTAGCATGCTGGGTAAACTGATTACAGGTGACGTGAAAC
TGAATAATCTGAGCGGTCCGATTAGCATTGCCAAAGGTGCAGGTATGACCGCAGAACTGGGCGTTGTTTAT
TACCTGCCGTTTCTGGCACTGATTAGCGTTAATCTGGGCATTATTAACCTGTTTCCACTGCCGGTTCTGGA
TGGTGGTCATCTGCTGTTTTTAGCCATTGAGAAAATCAAAGGTGGTCCGGTGAGCGAACGTGTTCAGGATT
TTTGTTATCGTATTGGTAGCATTCTGCTGGTTCTGCTGATGGGTTTAGCACTGTTTAATGATTTTAGCCGT
CTGGAATTTATCGAAGGTCGTCATCATCACCATCATCATATTGATGAAGAACAGAAACTGATCAGCGAAGA
AGATCTGCTGCGTAAACGT**TAATAA**CTCGAG

pTK1300 = pCOLADuet-1-*rseP-Myc H22F*
CC**ATG**GTGAGCTTTCTGTGGGATTTAGCCAGCTTTATTGTTGCACTGGGTGTTCTGATTACCGTGTTTGAA
TTTGGTCATTTTTGGGTTGCACGTCGTTGTGGTGTTCGTGTTGAACGTTTTAGCATTGGTTTTGGTAAAGC
ACTGTGGCGTCGTACCGATAAACTGGGCACCGAATATGTTATTGCACTGATTCCGTTAGGTGGCTATGTTA
AAATGCTGGATGAACGTGCAGAACCGGTTGTGCCGGAACTGCGTCATCATGCATTTAACAATAAAAGCGTT
GGTCAGCGTGCAGCAATTATTGCAGCAGGTCCGGTTGCCAATTTTATCTTTGCAATTTTTGCCTACTGGCT
GGTGTTTATTATCGGTGTTCCGGGTGTTCGTCCGGTTGTTGGTGAAATTGCAGCAAATAGCATTGCAGCCG
AAGCACAGATTGCACCGGGTACAGAACTGAAAGCAGTTGATGGTATTGAAACACCGGATTGGGATGCAGTT
CGTCTGCAGCTGGTTGATAAAATCGGTGATGAAAGCACCACCATTACCGTTGCACCGTTTGGTAGCGATCA
GCGTCGTGATGTTAAACTGGATCTGCGTCATTGGGCATTTGAACCGGATAAAGAAGATCCGGTTAGCAGCC
TGGGTATTCGTCCGCGTGGTCCGCAGATTGAACCGGTGCTGGAAAATGTTCAGCCGAATAGCGCAGCAAGC
AAAGCAGGTCTGCAGGCAGGCGATCGTATTGTGAAAGTGGATGGTCAGCCGCTGACACAGTGGGTTACCTT
TGTTATGCTGGTTCGTGATAATCCGGGTAAAAGCCTGGCACTGGAAATTGAACGTCAGGGTAGTCCGCTGA
GTCTGACCCTGATTCCGGAAAGCAAACCTGGTAATGGTAAAGCGATTGGCTTTGTGGGTATTGAACCGAAA
GTTATTCCGCTGCCGGATGAATATAAAGTTGTTCGTCAGTATGGTCCGTTTAACGCAATTGTTGAAGCAAC
CGATAAAACCTGGCAGCTGATGAAACTGACCGTTAGCATGCTGGGTAAACTGATTACAGGTGACGTGAAAC
TGAATAATCTGAGCGGTCCGATTAGCATTGCCAAAGGTGCAGGTATGACCGCAGAACTGGGCGTTGTTTAT
TACCTGCCGTTTCTGGCACTGATTAGCGTTAATCTGGGCATTATTAACCTGTTTCCACTGCCGGTTCTGGA
TGGTGGTCATCTGCTGTTTTTAGCCATTGAGAAAATCAAAGGTGGTCCGGTGAGCGAACGTGTTCAGGATT
TTTGTTATCGTATTGGTAGCATTCTGCTGGTTCTGCTGATGGGTTTAGCACTGTTTAATGATTTTAGCCGT
CTGGAATTTATCGAAGGTCGTCATCATCACCATCATCATATTGATGAAGAACAGAAACTGATCAGCGAAGA
AGATCTGCTGCGTAAACGT**TAATAA**CTCGAG

Supplementary Note 2: Peptide analysis of MBP-TNFα fusion proteins by LC-MS/MS

Trypsin digest was done by filter-aided sample preparation (FASP)[2] with some modifications: Proteins were denatured, reduced and alkylated on a 10K MWCO filter (PALL Corporation) and afterwards digested with 400 ng trypsin for 1h. 75 µl of the protein solution with 1 µg/µl were mixed with 500 µl 8 M urea and loaded onto the filters. After that, filters were washed once with 500 µl and spun down at 12,500 rpm in an Eppendorf centrifuge. Reduction and alkylation were performed in PreOmics LYSE buffer for 20 min at 52 °C (PreOmics, Munich). For digestion, 400 ng of modified trypsin (Promega) was added in 100 µl LYSE buffer. Peptide purification was performed according to the PreOmics iST protocol. RP (reversed phase) separation was done on a 1290 UHPLC (Agilent Technologies) equipped with an ACQUITY peptide CSH C18 column (2.1 x 150 mm, 1.7 µm, 130 Å; Waters Corporation) at 40 °C and 0.2 ml/min. Solvent A was 0.1% FA (formic acid) in water. Solvent B was 0.1% FA in acetonitrile. Total run time was 53 min, and the gradient was as follows. 0-8.0 min: 2% B, 8.0-38.0 min: linear increase from 2% to 40% B, 38.0-41.0 min: 90% B, 41.0-42.0 min: linear decrease from 90% to 2% B, 42.0-53.0 min: 2% B. MS analysis was performed on an Impact II ESI-QqTOF mass spectrometer (Bruker Daltonics) in auto MS/MS mode and positive polarity, in a mass range from 150 to 2200 m/z. The following ESI source parameters were used: end plate offset 500 V, capillary voltage 4500 V, nebulizer gas 2.0 bar, dry gas 8.0 l/min, dry temperature 250 °C. Auto MS/MS parameters are as follows: cycle time 2.0 sec, absolute threshold 500 cts per 1000 sum, Collision energy was 7eV. LC-MS/MS spectra were processed using PEAKS Studio X pro software[3] (PEAKS Studio 10.6 build 20201221, Bioinformatics Solutions, Canada). Peptides were evaluated with a precursor mass tolerance of 30 ppm and a MS/ MS fragment tolerance of 0.05 Da. Semi-specific trypsin specificity was selected, meaning that either the peptide N or C terminus was allowed a non-specific cleavage, maximum number of missed cleavages 4, and the following modifications: carbamidomethylation at Cys, monomethylation at Met, His Trp, dioxidation at Met, deamidation at Asn and Gln, pyro-Glu from Glu and Gln, internal disulfides at Cys, carbamylation at peptide N-termini, a maximum of 3 variable modifications were allowed per peptide. A custom database containing the TNFα protein was searched. Label-free quantification (LFQ) values for the listed peptides were manually extracted from the search result, and the percentage distribution in the sample was calculated (Supplementary Table 2).

Supplementary Note 3: MD simulations of RseP

We performed structure predictions of RseP wt (= RseP L2V) and of mutant RseP proteins, on a local installation of AlphaFold 2[4] with a database and source code install (https://github.com/deepmind/alphafold) dated March 30, 2022 on a dual AMD EPYC 7713 workstation with 265 logical cores and three NVIDIA RTX A5000 GPUs. The mandatory max_template_date parameter was set to 2022-12-01, model_preset monomer, all other input parameters as per default settings.

The obtained highest-rank structure prediction has a near-perfect overlap with the AlphaFold structure deposited in the Uniprot database (File: AF-P0AEH1-F1-model_v1.pdb). So, we proceeded with the modelling of the Zn^{2+} ion into the active site, which was placed manually to be coordinated by the side chains of H22, H26 and D402. This structure, containing the atom positions of the protein and the Zn^{2+} ion, was used as the starting structure for the input generator of the CHARMM-GUI[5] membrane builder algorithms.

In order to obtain MD trajectories which are independent of the initial configuration, the following workflow was done separately for the construction of six systems.

For membrane insertion and placement, we chose the PPM[6] 2.0 algorithm (https://opm.phar.umich.edu/ppm_server2), and chose to generate pore water and pore size based on protein geometry. To construct the system for MD simulations, we chose a rectangular box with a 2.25 nm minimum water height on top and bottom of the bilayer. Symmetric bilayers were constructed consisting of the following number of lipid molecules: 97 PMCL2 (cardiolipin lipid), 37 TYCL2 (cardiolipin lipid), 12 DPPE (1,2-Dipalmitoyl-*sn*-glycero-3-phosphoethanolamine), 12 DMPE (1,2-Dimyristoyl-*sn*-glycero-3-phosphoethanolamine), and 24 PYPE [1-hexadecanoyl-2-(9Z-hexadecenoyl)-*sn*-glycero-3-phosphoethanolamine]. Water molecules were added and the system was neutralized to zero charge by adding sodium and chloride ions to a final salt concentration of 100 mM (replacing water molecules; Supplementary Table 3). The CHARMM36m force field[7,8] was used to describe protein, lipids, and salt ions, while the TIP3P model was used for water[9]. For the production run, we employed the Parrinello-Rahman barostat[10] with a semi-isotropic pressure coupling scheme and a time constant set to 5.0 ps to maintain the pressure constant. The pressure was set to 1.0 bar and the isothermal compressibility to 4.5×10^{-5} bar^{-1}. The temperature was maintained at 310.15 K using the Nose-Hoover thermostat[11] with a time constant of 1.0 ps. Electrostatic interactions were handled using the PME method[12]. The cut-off length of 1.2 nm was used for electrostatic (real space component) and van der Waals interactions. Hydrogen bonds were constrained using the LINCS algorithm. Finally, periodic boundary conditions were applied in all directions. The simulations were carried out using an integration time step of 2 fs. Energy minimization, equilibration and production was performed on the Mahti supercomputer at the CSC - IT Center for Science, Finland. In total, six production runs for RseP wt were performed for a total of 2000 ns, employing GROMACS-2021 software[13].

RMSD analysis of the obtained trajectories showed a protein structure convergence after the first 500 ns. The first 250 nanoseconds of the resulting output trajectories were discarded and periodic boundary conditions were removed by centering the protein molecule in the system. To compute a representative structure of the system, the amino acid residues outlining the substrate binding pocket were defined: residues 38-44, 66-71, and 425-439. This group of residues was now used to compute an RMSD-based clustering of the trajectory with a cut-off distance of 0.15 nm by the GROMOS algorithm[14]. Around 40 clusters were found for the RseP

wt system. The middle structure of the main cluster was then chosen to generate a representative image of the protein and its embedding into the membrane bilayer.

Supplementary References

1. Kanehara, K., Akiyama, Y. & Ito, K. Characterization of the *yaeL* gene product and its S2P-protease motifs in *Escherichia coli*. *Gene* **281**, 71-79 (2001).
2. Wisniewski, J. R., Zougman, A., Nagaraj, N. & Mann, M. Universal sample preparation method for proteome analysis. *Nat. Methods* **6**, 359-362 (2009).
3. Ma, B. *et al.* PEAKS: powerful software for peptide de novo sequencing by tandem mass spectrometry. *Rapid Commun. Mass Spectrom.* **17**, 2337-2342 (2003).
4. Jumper, J. *et al.* Highly accurate protein structure prediction with AlphaFold. *Nature* **596**, 583-589 (2021).
5. Lee, J. *et al.* CHARMM-GUI Input Generator for NAMD, GROMACS, AMBER, OpenMM, and CHARMM/OpenMM Simulations Using the CHARMM36 Additive Force Field. *J. Chem. Theory Comput.* **12**, 405-413 (2016).
6. Lomize, M. A., Pogozheva, I. D., Joo, H., Mosberg, H. I. & Lomize, A. L. OPM database and PPM web server: resources for positioning of proteins in membranes. *Nucleic Acids Res.* **40**, D370-376 (2012).
7. Best, R. B. *et al.* Optimization of the additive CHARMM all-atom protein force field targeting improved sampling of the backbone ϕ, ψ and side-chain χ_1 and χ_2 dihedral angles. *J. Chem. Theory Comput.* **8**, 3257-3273 (2012).
8. Huang, J. *et al.* CHARMM36m: an improved force field for folded and intrinsically disordered proteins. *Nat. Methods* **14**, 71-73 (2017).
9. Jorgensen, W. L., Chandrasekhar, J., Madura, J. D., Impey, R. W. & Klein, M. L. Comparison of simple potential functions for simulating liquid water. *J. Chem. Phys.* **79**, 926-935 (1983).
10. Parrinello, M. & Rahman, A. Polymorphic transitions in single crystals: A new molecular dynamics method. *J. Appl. Phys.* **52**, 7182-7190 (1981).
11. Hoover, W. G. Canonical dynamics: Equilibrium phase-space distributions. *Phys. Rev. A* **31**, 1695-1697 (1985).
12. Essmann, U. *et al.* A smooth particle Ewald method. *J. Chem. Phys*. **103**, 8577-8593 (1995).
13. Abraham, M. J. *et al.* High performance molecular simulations through multi-level parallelism from laptops to supercomputers. *SoftwareX* **1-2**, 19-25 (2015).
14. Daura, X. *et al.* Peptide Folding: When simulation meets experiment. *Angew. Chem. Int. Ed*. **38**, 236-240 (1999).
