# Supplementary material for: In vivo characterization of the bacterial intramembrane-cleaving protease RseP using the heme binding tag-based assay iCliPSpy
Source: Commun Biol. 2023 Mar 18;6:287. doi: 10.1038/s42003-023-04654-z (PMC10024687; doi:10.1038/s42003-023-04654-z)
Supplement: Supplementary file 3 — Description of Additional Supplementary Files [file 42003_2023_4654_MOESM3_ESM.pdf]

Description of Additional Supplementary Files

File name: Supplementary Data 1

Description: The source data behind the graphs (Figures 2f, 4c-e, 5c) in the paper
